# Duration of motherhood has incremental effects on mothers’ neural processing of infant vocal cues: a neuroimaging study of women

**DOI:** 10.1038/s41598-017-01776-3

**Published:** 2017-05-11

**Authors:** Christine E. Parsons, Katherine S. Young, Mikkel V. Petersen, Else-Marie Jegindoe Elmholdt, Peter Vuust, Alan Stein, Morten L. Kringelbach

**Affiliations:** 1 Interacting Minds Center, Department of Clinical Medicine, Aarhus University, Aarhus C, Denmark; 2 Center for Music in the Brain (MIB), Department of Clinical Medicine, Aarhus University, DK & The Royal Academy of Music, Aarhus/Aalborg, Denmark; 3 Department of Psychiatry, University of Oxford, Oxford, OX3 7JX UK; 4 Anxiety and Depression Research Center, Department of Psychology, UCLA, Los Angeles, CA USA; 5 Center of Functionally Integrative Neuroscience, Aarhus University Hospital, DK Aarhus C, Denmark; 6 Institut d’études avancées de Paris, Paris, France; 7 MRC/Wits Rural Public Health and Health Transitions Research Unit (Agincourt), School of Public Health, Faculty of Health Sciences, University of the Witwatersrand, Johannesburg, South Africa

## Abstract

The transition to motherhood, and the resultant experience of caregiving, may change the way women respond to affective, infant signals in their environments. Nonhuman animal studies have robustly demonstrated that mothers process both infant and other salient signals differently from nonmothers. Here, we investigated how women with and without young infants respond to vocalisations from infants and adults (both crying and neutral). We examined mothers with infants ranging in age (1–14 months) to examine the effects of duration of maternal experience. Using functional magnetic resonance imaging, we found that mothers showed greater activity than nonmothers to vocalisations from adults or infants in a range of cortical regions implicated in the processing of affective auditory cues. This main effect of maternal status suggests a general difference in vocalisation processing across infant and adult sounds. We found that a longer duration of motherhood, and therefore more experience with an infant, was associated with greater infant-specific activity in key parental brain regions, including the orbitofrontal cortex and amygdala. We suggest that these incremental differences in neural activity in the maternal brain reflect the building of parental capacity over time. This is consistent with conceptualizations of caregiving as a dynamic, learning process in humans.

## Introduction

Becoming a mother brings about profound changes in the female mammalian brain. These changes are assumed to be necessary and adaptive in supporting sensitive, responsive caregiving. Much of our understanding about the impact of motherhood derives from nonhuman animal studies, which provide robust evidence for brain plasticity during pregnancy, extending into the postpartum period and beyond (for recent reviews, see refs 1, 2). Key structures underpinning rodent maternal care include the medial pre-optic area of the hypothalamus, projecting to the meso-limbic dopamine circuits and the amygdala, which support initiation of maternal behavior, maternal motivation and vigilance for infant safety respectively^3^. In humans, functional magnetic resonance imaging (fMRI) studies of adults’ responses to infant cues have highlighted a similar subcortical network^4^, indicating the conserved nature of maternal behavior^5^. There is also mounting evidence that human caregiving responses additionally rely on cortical regions important for social cognition and reward, including the anterior cingulate cortex (ACC), the superior temporal gyrus, and sulci (STG/STS) and the orbitofrontal cortex (OFC)^2^. This may be related to cross-specifies differences in maternal behavior, such as the extended and complex caregiving demands of highly dependent human infants^6, 7^.

Motherhood and caregiving are considered to bring about fundamental changes in how females perceive and react to infants. However, a growing body of studies indicates that the transition to motherhood may also be associated with alterations to the way females process their broader environment (for review, see ref. 8). It has long been argued that early motherhood involves an emotional rebalancing that moderates reactivity to negative or aversive environmental events, while simultaneously promoting attention to infant signals and the ability to attend sensitively to infant needs^9, 10^. Again, this has been extensively studied in laboratory rats. Typically, early postpartum females show reduced fear and anxious behaviors compared to nulliparous females, greater resilience to stress, and better memory^11–14^.

While these behavioural changes do not directly mediate caregiving, they create conditions in which the female is more motivated to explore the environment, better able to source food, and generally organize her behaviour towards her infant. Studies on human mothers are less conclusive. Many women experience decreased anxiety after giving birth, and an increase in positive mood (for reviews, see refs 15, 16). However, there is a significant subpopulation of women who experience significant emotional dysregulation during the first postpartum weeks or months^17, 18^. Furthermore, findings on memory and cognitive changes in human mothers have also been mixed, and not entirely consistent with animal studies showing positive effects^16, 19^. Such discrepancies between nonhuman and human studies highlight the need to investigate the extent to which functional changes in emotional processing occur in human motherhood.

A small number of studies have sought to determine the structural and functional brain changes associated with motherhood in humans. One study reported an increase in mothers’ gray matter volume in the prefrontal cortex, parietal lobes and midbrain areas across the initial months postpartum^20^. Studies of neural activity measuring event-related potentials have typically demonstrated enhanced processing of infant-specific signals, such as facial expressions using event-related potentials^21, 22^. One fMRI study found that mothers showed greater activity in the amygdala and limbic regions to infant crying, but non-mothers showed greater activity to infant laughter^23^.

Studies of nonhuman animals offer the intriguing hypothesis that maternal status and caregiving experience in general has a broad impact on neural processes. To examine this, we compared women with and without young infants responding to distress and neutral vocalisations from both adults and infants using fMRI. Therefore, our focus was not on describing neurobiological changes occurring before and after birth, but rather on whether the duration of caregiving experience with an infant has an effect on maternal brain functioning. Furthermore, the majority of studies to date examining vocal cues have focused on responses to infant crying. For instance, a number of studies have examined responses to familiar and unfamiliar crying^24, 25^, infant crying of different distress levels^26^ or to infant crying compared to white noise (for review see, refs 24, 27). However, less is known about how we differentiate infant from adult crying, or indeed adult from infant vocal sounds more broadly. Taking crying specifically, both infant and adult cry sounds are powerful signals of conspecific distress. However, the typical listener response is different^28^. Listening to infant crying can facilitate faster, effortful motor responses than adult crying^29, 30^ and is associated with a greater reported motivation to respond^31^. Infant crying is clearly powerful in initiating maternal care, but other infant vocal emotional signals, such as babbling, emerge early in life and also engage and motivate caregivers. One of the few studies comparing responses to adult and infant non-cry sounds reported that infant ‘babble’ sounds elicited greater ‘motivation to respond’ ratings than the equivalent adult neutral sounds^31^. Here, we use both cry and neutral sounds from adults and infants, in order to investigate how we differentiate infant from adult sounds generally.

Furthermore, within mothers, we investigated the effect of experience-dependent plasticity, by testing mothers with infants of a range of ages. By examining motherhood as a continuum of experience, we advance previous studies which assessed whether motherhood has a categorical effect on responding to infant cues, by investigating overall group differences. We further speculated, based on previous animal studies and reviews of human literature (e.g., refs 32–34) that mothers would differ from non-mothers in responding to emotional cues overall. We expected that mothers would show heightened activity in response to infant vocalisations compared to non-mothers. These differences were expected to occur in emotion regulation regions (e.g., OFC, dlPFC, MFG, frontopolar cortex, as reviewed^35^). We also explored whether responding to infant cues would vary by the duration of maternal experience.

## Methods

### Participants

Participants were recruited from the general community in Aarhus, Denmark, using posters, online advertisements, and social media. Inclusion criteria for participation were: not currently experiencing any psychological or physical conditions, not taking psychotropic medication, no problems with hearing, normal vision or vision corrected to normal. Participants were largely drawn from the Aarhus University staff and student populations. Fifty-eight women (n = 29 mothers) participated, aged between 22 and 35 years (M = 27.93, SD = 3.24). The age range of the mothers’ infants was between 1 and 14 months (M = 8.41 months, SD = 3.36). The majority of mothers were primiparous (23 mothers), but 6 mothers had two children. There were no significant differences between the mothers and non-mothers scores on the Becks Depression Inventory, the State and Trait Anxiety Questionnaire and the Empathy Quotient (see Table 1).

**Table 1:** Participant demographics by parental status.

Measure	Mothers (n = 29) Mean (SD)	Non-mothers (n = 29) Mean (SD)	Statistics (independent samples t-test)
Age	29.41 (3.37)	26.45 (2.37)	p < 0.0001
Beck Depression Inventory	4.28 (3.58)	3.36 (2.97)	p = 0.28
State Anxiety	26.65 (6.27)	28.38 (5.63)	p = 0.27
Trait Anxiety	30.00 (8.23)	32.59 (6.89)	p = 0.20
Empathy Quotient Scores	53.24 (9.56)	50.62 (7.44)	p = 0.63
Years of Music Training	2.59 (4.30) (n = 11)	3.36 (4.82) (n = 14)	p = 0.43

The mothers were on average, significantly older than the non-mothers, by about 3 years. Ten of the mothers were currently breastfeeding. Twenty-three of the mothers had standard births and 6 had caesarean section births. Eleven of the mothers and 14 of the non-mothers reported having some music training. The two groups reported similar years of music training. The majority of the sample (32/57) reported earning salaries of over 20.000 Danish kroner per month (approx. € 2,700/month). Mothers reported higher salaries overall compared to non-mothers (N = 57; Chi square comparing salary categories; df(4) = 20.36; p = 0.001). All mothers reported being either married or co-habiting, whereas for the non-mothers, 17 were married/cohabiting and 12 were single (Chi square; df(2) = 29.79; p = 0.001).

### Ethical statement

All methods were carried out in accordance with the Helsinki Declaration. Ethical approval for the study was granted by the Committee of Central Region Denmark. All participants provided written informed consent for participation.

### Experimental stimuli and task

Stimuli consisted of adult and infant vocalisations, categorized as ‘distressed’ (cries) or ‘neutral’ (i.e., infant babbling, or for adults, sounds such as ‘emm’). Twelve examples of each sound category were taken from a standardized database, the “Oxford Vocal Sounds Database” described in detail elsewhere^31, 36^. Briefly, infant stimuli were taken from video recordings of infants interacting with their at home (see ref. 37). Infants were all full-term, healthy, and aged between 6 and 8 months at the time of recording (*M* = 6.7 months, *SD* = 0.9). Adult stimuli were obtained from video diary blogs recorded by females, aged around 18–30 years (see ref. 31 for details). Stimuli were presented using Presentation^®^ software (Neurobehavioral Systems, Inc.).

Stimuli were presented in blocks of four sounds from the same category (adult cry, infant cry, adult neutral or infant neutral). Each individual stimulus was 1500 ms in duration and was presented with a 250 ms (within-block) inter-stimulus interval. The inter-block interval was 750 ms. Each block was repeated eighteen times so that participants heard 72 sounds from each category (e.g., 72 infant cries). Blocks of silence, also of nine-second duration, were repeated 54 times in total. Presentation of stimuli within each block and block order was randomized across participants. Table 2 presents the physical features of the infant and adult stimuli. There were no significant differences between the adult and infant stimuli in terms of fundamental frequency (*F*_0_) as indicated by independent t-tests, but the adult stimuli had shorter burst durations than the infant stimuli.

**Table 2:** Physical parameters of vocalization stimuli.

	Infant vocalisations		Adult vocalisations		*t*-value	*P*-value
	M (SD)	Range	M (SD)	Range		
*F*_0_ (Hz)	376.06, 82	257.81–527.56	334.77, 94.59	175.78–574.22	1.81	0.08
Burst duration (s)	1, 0.44	0.28–1.50	0.72, 0.24	0.23–1.5	3.2	0.002
Number of bursts	1.73, 1.76	1–4	2.13, 1.58, 1.56	1–3		

*F*_0_, fundamental frequency.

fMRI scanning and analysis.

Sounds were presented at a comfortable hearing level, assessed prior to the onset of scanning. Participants were asked to press a button on hearing a tone (400 Hz, 500 ms). This was to ensure sustained attention to the sound stimuli. The tones were played 4 times over the entire task. The total task time was 18.9 mins and was presented using MRI-compatible headphones.

Structural and functional MRI data were collected on a Siemens 3 T Trio scanner with a 32-channel head coil. A T1-weighed structural image was acquired for each participant (176 slices, voxel size = 1 × 1 × 1 mm3, matrix size = 256 × 246, TE = 2.52 ms, TR = 1900 ms, flip angle = 9°, interslice gap = 3 mm). Functional data, consisting of 756 volumes, were acquired in one experimental session. T2*-weighted, echoplanar images were acquired using an interleaved slice acquisition (TR, 1500 ms; TE, 27 ms; flip angle, 70°; 30 slices of 3 mm thickness; in-plane resolution, voxel size = 3 × 3 × 3 mm3, FOV, 192 × 192 mm, interslice gap = 3 mm). Soft cushions were used to minimize head movement.

Pre-processing of data was carried out using SPM12 (Wellcome Trust Center for Neuroimaging, University College London, UK, http://www.fil.ion.ucl.ac.uk). Functional images for each participant were slice-time corrected, realigned and unwarped to correct for head motion, co-registered to the structural images, normalized into a standard stereotactic space as defined by the Montreal Neurological Institute and smoothed with an 8 mm Gaussian kernel, full width at half maximum. Using the GLM Flex toolbox for Matlab (version: GLM_Flex2; http://mrtools.mgh.harvard.edu/index.php?title=GLM_Flex), a 2 × 2 mixed design ANOVA was conducted with one between subjects’ factor, maternal status (mother, nonmother) and one within-subjects’ factor, stimulus age (infant, adult; collapsing across cry and neutral sounds).

We used the infant age (months) for each primiparous mother as a measure of duration of current experience with an infant, entering it as a covariate of interest for the contrasts differentiating infant from adult vocalisations. The rationale for selecting this contrast was that mothers are likely to accumulate greater listening experience with infant sounds over adult sounds than nonmothers. We performed this analysis with primiparous women only because infant age directly relates to extent of maternal experience (N = 23). Whole-brain corrections for multiple comparisons were implemented using bspmview (cluster-corrected threshold of FWE; *p* < 0.05).

## Results

**Figure 1:**
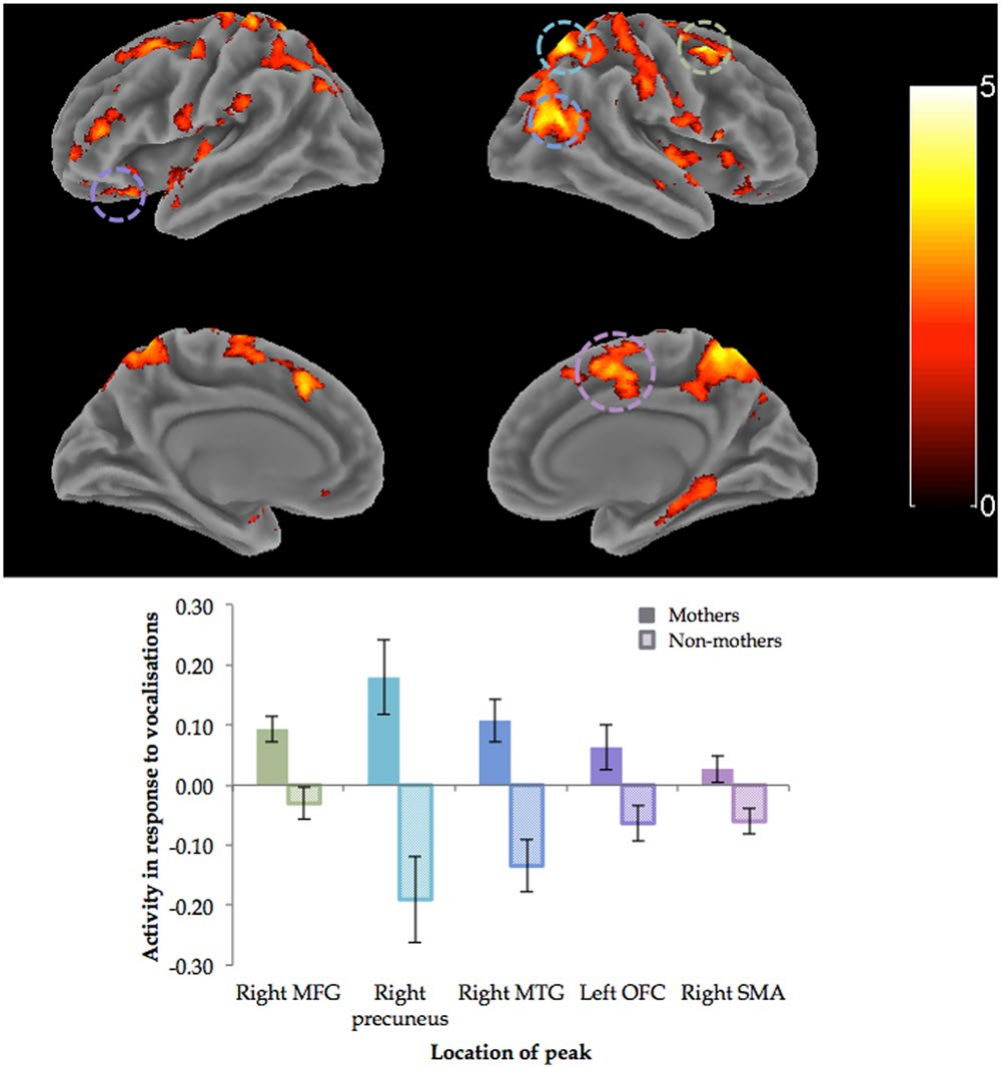
Contrasting brain activity in mothers and non-mothers: Mothers demonstrated greater reactivity to vocalisations from infants and adults across a range of cortical regions implicated in the processing of affective auditory cues. These regions included areas involved in executive functions and higher order cognitive functions (middle frontal gyrus; MFG; precuneus)^56^, interpretation of vocal content (middle temporal gyrus, MTG), affective processing (orbitofrontal cortex, OFC) and preparatory motor responses (supplementary motor area, SMA). [Upper: whole brain images are displayed with a *p* < 0.03, FWE-cluster corrected for multiple comparisons for ease of visualisation; Lower: parameter estimates were extracted from 6 mm radius spheres centered on peak coordinates identified in the whole brain GLM. Error bars represent mean+/− standard error].

Table 3 presents the results of a GLM, examining the main effects of maternal status (mother, nonmother) and stimulus age (infant, adult) and their interaction. For the main effect of maternal status, mothers demonstrated greater activity compared to nonmothers in an extended region with peaks in the middle frontal gyrus, middle temporal gyrus, precuneus, the orbitofrontal gyrus and the supplementary motor area (main peak co-ordinates presented in Table 3). Figure 1 presents the main effects of parental status, extracting data from 6 mm ROI spheres around key peaks in the significant cluster. We also examined these ROIs using data from first time mothers only (N = 23), and found the same pattern of significant effects (all *p*’s < 0.05). Comparing nonmothers > mothers, none of the effects survived multiple comparison correction.

**Table 3:** Examining the effects of maternal status, stimulus age and their interaction across all participants (N = 58). Reporting peak MNI co-ordinates within clusters, applying cluster FWE correction (p < 0.05). Labelling of peaks within the larger significant clusters was done using the AAL2 atlas in bpsmview.

Contrast	Region	Cluster size	T – stat	x	y	z
Mothers > nonmothers	Right middle frontal gyrus	32413	5.14	34	4	58
	Right middle frontal gyrus	32413	4.08	28	14	44
	Right precuneus	32413	4.87	8	−62	66
	Superior parietal lobule	32413	4.68	18	−58	54
	Right middle temporal gyrus	32413	4.63	44	−64	20
	Left superior temporal pole	32413	4.25	−32	18	−26
	Left orbitofrontal cortex	32413	4.24	−28	34	−18
	Right supplementary motor area	32413	4.11	12	4	50
Adult > infant	Left middle frontal gyrus	1539	−3.61	−38	44	30
	Left middle frontal gyrus	1539	−3.43	−46	30	34
Maternal status X age	Right inferior occipital gyrus	307	15.00	40	−82	−12
	Right lingual gyrus	307	14.68	18	−86	−12
	Right middle occipital gyrus	307	7.54	36	−94	0

Effects of infant age on maternal brain response.

For the main effect of stimulus age, adult vocalisations were associated with greater activity than infant vocalisation in a region peaking in the middle frontal gyrus (see Table 3). Comparing infant vocalisations > adult vocalisations, none of the effects survived multiple comparison correction. For the interaction effect between maternal status and stimulus age, significant differences emerged for a region of the occipital lobe. Due to scanning parameters related to slice angle, we did not obtain full coverage of the occipital region for all participants. To fully describe our GLM, we present the results of this interaction, but we did not explore it further.

**Figure 2:**
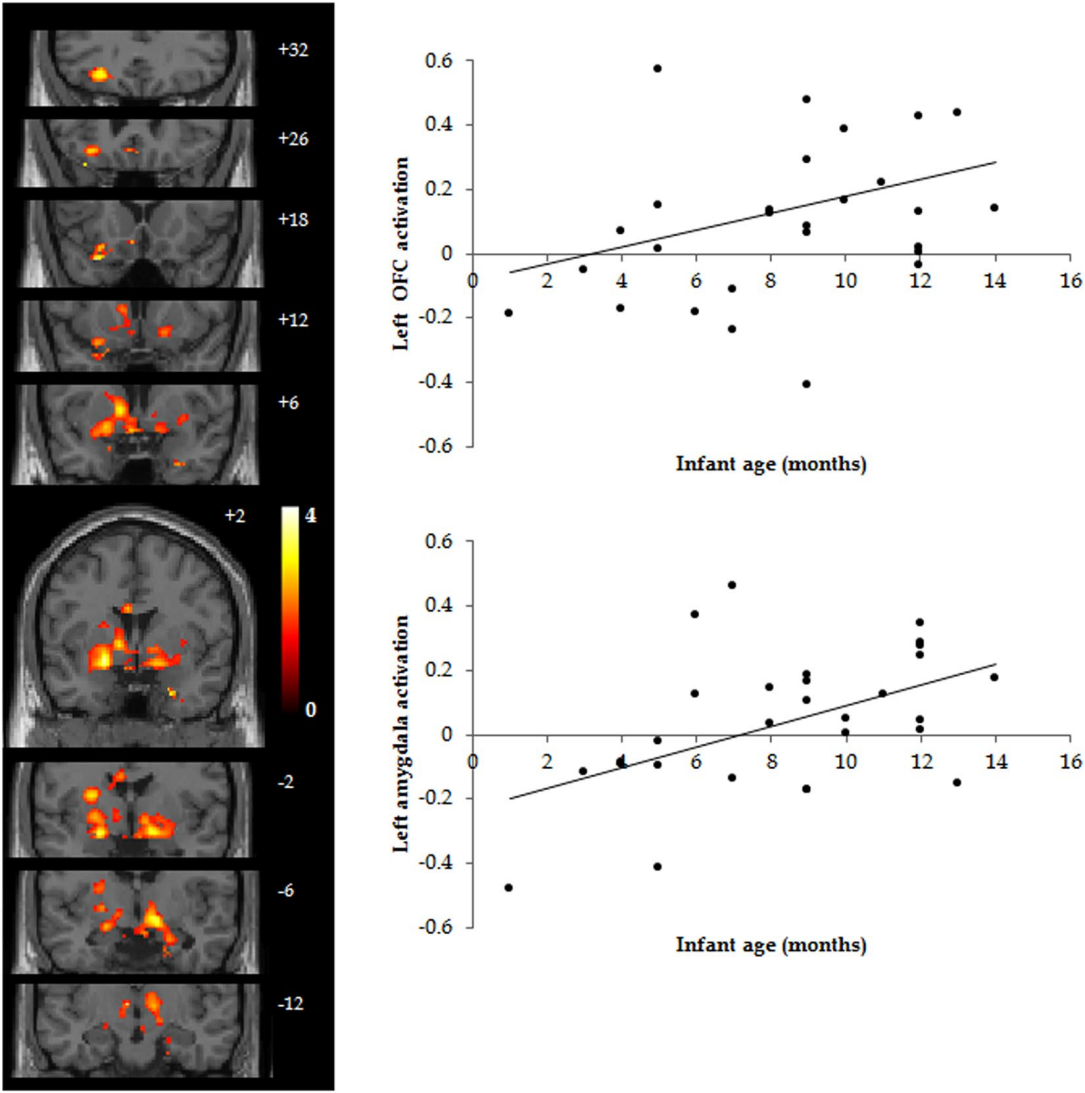
Motherhood has a ‘dose-dependent’ effect on the processing of basic infant emotional signals. Infant age was significantly correlated with mothers’ differential reactivity to infant and adult vocalisations in a cluster encompassing left amygdala and left OFC (main effect of age; infant > adult vocalisations; regression analysis; p < 0.05, cluster-FWE correction). Scatter plots display parameter estimates from significant clusters for left OFC (upper) and left amygdala (lower). These regions of interest were defined as 6 mm-radius spheres centered on peak coordinates identified in the whole-brain analysis.

Table 4 presents the results of a whole brain regression analysis comparing differences in neural activity related to infant age (in months, primiparous mothers only). Across first time mothers (N = 23), for the infant vocalization > adult vocalization contrast, infant age was positively associated with increased activity peaking in a number of regions including the parahippocampal gyrus, orbitofrontal cortex and the amygdala (major peaks listed in Table 4). Figure 2 presents scatterplots of the relationship between infant age and the left amygdala and left OFC activity. Infant age, in months, was positively correlated with activity in these regions. For the adult > infant vocalization contrast, no effects survived multiple comparison correction.

**Table 4:** Effects of duration of motherhood: regression analysis using infant age in months, applying cluster FWE correction (p < 0.05), across first-time mothers (N = 23). Labelling of peaks within the larger significant clusters was done using the AAL2 atlas in bpsmview.

Contrast	Region	Cluster size	T – stat	x	y	z
Infant > Adult	Thalamus	2097	4.42	−10	−4	2
	Left parahippocampal gyrus	2097	4.34	−28	−8	−32
	Left amygdala	2097	3.88	−20	−2	−14
	Left inferior frontal gyrus, pars triangularis	2097	3.70	−52	32	24
	Left middle temporal pole	2097	3.54	−44	10	−26
	Left orbitofrontal cortex	2097	3.48	−30	18	−24

## Discussion

Our key finding is that a longer duration of motherhood, and therefore extended experience with an infant, is associated with greater infant-specific activity in key parental brain regions, including the OFC and amygdala. We also found differences between mothers and non-mothers in responses to emotional vocalisations overall, from adults or infants. Mothers showed greater activity in sensory auditory regions, namely the MTG, and parietal and temporal regions compared to non-mothers. Overall, across all participants, adult vocalisations elicited greater activity compared to infant vocalisations in a frontal lobe region, with a peak in the left middle frontal gyrus.

The finding that extended experience with an infant heightens differential responding to infant vocalisations is consistent with the conceptualization of caregiving as a dynamic, learning process in humans^35^. These increased responses with extended experience were found in regions referred to as the “emotional” components of the caregiving system (and not the entire caregiving brain network, as described by ref. 32) namely the amygdala and the OFC. There is limited behavioural evidence of how experience changes mothers’ responses to infants’ auditory cues. For crying specifically, one study suggests that with growing experience, mothers acquire a more differentiated understanding of their infant’s crying and ability to manage their own responses^38^. The idea that mothers’ responses to infant vocalisations may be altered through experience is intuitive when considering mothers’ auditory environments. For example, infants from birth to 3 months are estimated by their mothers to cry for about 121 minutes per day^39^. Mothers accumulate extended listening experience with infant vocal cues, which may shape their neural responses to these sounds, as musical training shapes responses to music stimuli^40^.

The functional differences in the amygdala and OFC found here, related to duration of motherhood, mirror the structural changes reported in a longitudinal study of postnatal gray matter increases^20^. In the Kim *et al*. study, mothers were tested early postpartum (at 2–4 weeks) and again at 3 to 4 months. Increases in amygdala gray matter, along with other subcortical structures, were correlated with mother’s positive perceptions of their infants. Two cross-sectional fMRI studies of women have shown differences in amygdala responses to infant cues dependent on mothers’ own attachment experiences (not measured in this study). In one study comparing adults’ own attachment experiences, insecurity was associated with greater activity in right amygdala to infant crying^41^. In another study examining mothers’ attachment towards their own infant, more positive attachment feelings were associated with greater amygdala activity, when comparing responses to the mothers’ own infants’ positive face compared to an unfamiliar infants’^42^.

More broadly, amygdala nuclei are known to mediate emotional learning, increasing attention to, and arousal for, emotionally-salient cues^43^. The amygdala may therefore underlie parental vigilance for infant signals and motivation to respond^35^. These nuclei are bidirectionally coupled with the mPFC, a feature thought to facilitate regulation of emotional responses^44–46^. It has been suggested that maternal amygdala activity, which occurs in response to salient emotional cues, may be part of the neural circuitry that supports attunement to infant emotional states (e.g., ref. 47). Other conceptualisations of the parental brain have emphasized the amygdala as part of memory processing circuitry, together with the hippocampus and parahippocampus^34^. Given these suggested roles, together with the growing number of fMRI studies, the amygdala appears to be crucial in parental brain circuitry, and experience-dependent changes therein.

Like the amygdala, the orbitofrontal cortex has also been repeatedly demonstrated as a central node in the parental brain (for reviews, see refs 27, 48). Processing in the OFC is fundamental for the appraisal of emotional expressions across modalities^49–51^. The OFC effects found here for mothers with greater infant experience may be associated with enhanced appraisal processes, important for parental behaviour. However, further work relating experience with an infant, maternal sensitivity and OFC activity would be required to explore this.

We found overall differences between the processing of emotional vocalizations in mothers and nonmothers, in regions implicated in executive functions (middle frontal gyrus), interpretation of vocal content (middle temporal gyrus), affective processing (orbitofrontal cortex, OFC) and preparatory motor responses (supplementary motor area, SMA). This overall difference in auditory emotion processing is consistent with findings from nonhuman animal studies showing general changes in emotion processing with motherhood (for review, see ref. 1). A recent longitudinal study of women before pregnancy and at two times points post-pregnancy provided convincing evidence for lasting grey matter volume changes in brain regions involved in social processes^52^. Human motherhood might therefore have a widespread impact on responding to salient emotional information from the environment. However, given the cross-sectional nature of this study, it would be helpful to examine women longitudinally at different stages of motherhood to identify when such differences potentially emerge.

### Limitations

We used an incidental listening measure as in previous magnetoencephalography work^53^, which was designed to mimic how adults often encounter infant vocalisations. For instance, infant crying frequently occurs when parents are engaged in another task and the sound functions to draw the adult to the infant^54^. However, we cannot directly relate the patterns of neural processing found here to actual differences in maternal behavioural sensitivity to the infant. Future work might combine observations of mother-infant interactions with neural measures of maternal responses to infant cues.

We aimed to test a relatively large sample of women with and without young infants, but our sample was not homogeneous. Mothers differed on their breastfeeding status and mode of infant delivery, and mothers were three years older and had higher incomes than non-mothers.

Additional variables that may account for individual differences in maternal response to infant cues include current and planned caregiving role, personality traits and own attachment history. Future work might helpfully take account of these variables. Finally, other studies have asked parents about the nature and extent of their caregiving roles^55^. We relied on a different measure, infant age, to explore the extent of current experience with an infant. A combination of these two measures may provide a more complete measure of actual and perceived caregiving experience.

## Conclusions

Consistent with findings from other species, we demonstrate that motherhood is associated with robust changes in women’s neural responses to emotional cues, both infant-specific and more broadly. Furthermore, we demonstrate that the duration of motherhood has an impact on the neural processing of infant-specific cues in a dose-dependent fashion: greater experience is associated heightened, differential reactivity. We argue that these incremental changes in activity in several parental brain regions, including the amygdala and OFC, reflect the building of parental capacity over time, as the mother’s experience of caregiving increases. This suggests a critical role of learning in shaping neural responses implicated in caregiving.
